# Relationship between Plasma Analytes and SPARE-AD Defined Brain Atrophy Patterns in ADNI

**DOI:** 10.1371/journal.pone.0055531

**Published:** 2013-02-08

**Authors:** Jon B. Toledo, Xiao Da, Priyanka Bhatt, David A. Wolk, Steven E. Arnold, Leslie M. Shaw, John Q. Trojanowski, Christos Davatzikos, Alzheimer’s Disease Neuroimaging Initiative

**Affiliations:** 1 Department of Pathology & Laboratory Medicine, Institute on Aging, Center for Neurodegenerative Disease Research, University of Pennsylvania School of Medicine, Philadelphia, Pennsylvania, United States of America; 2 Section of Biomedical Image Analysis, Department of Radiology, University of Pennsylvania, Philadelphia, Pennsylvania, United States of America; 3 Penn Memory Center, University of Pennsylvania, Philadelphia, Pennsylvania, United States of America; Institute Biomedical Research August Pi Sunyer (IDIBAPS) - Hospital Clinic of Barcelona, Spain

## Abstract

Different inflammatory and metabolic pathways have been associated with Alzheimeŕs disease (AD). However, only recently multi-analyte panels to study a large number of molecules in well characterized cohorts have been made available. These panels could help identify molecules that point to the affected pathways. We studied the relationship between a panel of plasma biomarkers (Human DiscoveryMAP®) and presence of AD-like brain atrophy patterns defined by a previously published index (SPARE-AD) at baseline in subjects of the ADNI cohort. 818 subjects had MRI-derived SPARE-AD scores, of these subjects 69% had plasma biomarkers and 51% had CSF tau and Aβ measurements. Significant analyte-SPARE-AD and analytes correlations were studied in adjusted models. Plasma cortisol and chromogranin A showed a significant association that did not remain significant in the CSF signature adjusted model. Plasma macrophage inhibitory protein-1α and insulin-like growth factor binding protein 2 showed a significant association with brain atrophy in the adjusted model. Cortisol levels showed an inverse association with tests measuring processing speed. Our results indicate that stress and insulin responses and cytokines associated with recruitment of inflammatory cells in MCI-AD are associated with its characteristic AD-like brain atrophy pattern and correlate with clinical changes or CSF biomarkers.

## Introduction

Alzheimeŕs disease (AD) is defined by extracellular deposits of Aβ in senile plaques and intracellular aggregates of tau protein in neurofibrillary tangles accompanied by neuronal loss [1], [2], [3], [4], [5] in association with other abnormalities such synaptic and dendritic loss [6], [7], [8], [9], inflammation [10], [11], [12] and gliosis [13]. However, it is increasingly evident that these pathologies slowly emerge over a decade or more before AD is diagnosed clinically [14] and progresses through different pathophysiological stages that ultimately culminate in death [15].

Genetic heritability accounts for 60–80% of the risk for AD [16], with the APOE ε4 allele being the major genetic risk factor for AD in a dose dependent manner. Environmental factors and vascular risk factors such as head trauma, metabolic syndrome, education, hypertension, diabetes, stress, etc. [17] also increase the risk for AD, and it is postulated that changes in life style practices could reduce the risk for AD [18]. For example, vascular risk factors may cause cognitive changes via different but inter-related pathways, which converge to induce cerebrovascular pathology and Aβ deposition in brain vasculature [19], [20], [21].

The availability of neuroimaging biomarkers to monitor and track morphological brain changes and multi-panel molecular biomarkers that reflect different inflammatory and other biochemical pathways enable dissection and investigation of pathways that may be related to brain atrophy and pathology in patients with neurodegenerative disease.

To investigate how blood-based biochemical biomarkers may relate to AD specific brain atrophy, we chose to use an index known as SPARE-AD (Spatial Pattern of Abnormality for Recognition of Early Alzheimer’s disease) that maximally captures spatial patterns of brain atrophy related to AD, and which may be more sensitive than a single region of interest, such as hippocampal volume [22], [23]. Positive SPARE-AD values at baseline have also been associated with subsequent cognitive decline and conversion from mild cognitive impairment (MCI) to AD [24], [25], whereas SPARE-AD values have been found to increase with age and to correlate with cognitive performance in cognitively normal older adults [26].

We tested the association of 130 plasma analytes measured simultaneously using a large-scale commercial multiplex panel (Rules Based Medicine (RBM) Inc. (Austin, TX)) with the SPARE-AD to identify analytes related to disease pathways or specific patterns of structural changes in AD patients.

## Methods

### Subjects

Data used in the preparation of this article were obtained from the Alzheimer’s Disease Neuroimaging Initiative (ADNI) database (adni.loni.ucla.edu). The ADNI was launched in 2003 by the National Institute on Aging, the National Institute of Biomedical Imaging and Bioengineering (NIBIB), the Food and Drug Administration, private pharmaceutical companies and non-profit organizations. Its primary goal has been to test whether serial magnetic resonance imaging (MRI) [27], positron emission tomography (PET) [28], other biological markers [29], and clinical and neuropsychological assessment [30] can be combined to measure the progression of MCI and early AD. The Principal Investigator of this initiative is Michael W. Weiner, MD, VA Medical Center and University of California – San Francisco. ADNI is the result of efforts of many co- investigators from a broad range of academic institutions and private corporations, and subjects have been recruited from over 50 sites across the U.S. and Canada. At baseline, all subjects scored 6 or less in the short version of the geriatric depression scale (GDS-15) [31], which excludes subjects with depression. Exclusion criteria included any serious neurological disease other than possible AD, any history of brain lesions or head trauma, any recent history of substance abuse, any significant systemic illness or unstable medical condition and a previous history of major depression, bipolar disorder or schizophrenia. At baseline patients were not taking psychoactive medication (including antidepressants, neuroleptics, chronic anxiolytics or sedative hypnotics). For more details, see http://www.adni-info.org. Data was downloaded March 2012.

We included 818 adult subjects who had at least an MRI, 55 to 90 years old, who meet criteria for a clinical diagnosis of mild cognitive impairment (MCI, n = 396), probable AD (n = 193) or cognitively normal (CN, n = 229). Of these subjects, 58 CN, 395 MCI and 112 AD subjects had plasma RBM Luminex results. Subjects were also classified based on the presence of a normal or pathological/AD cerebrospinal fluid (CSF) signature using the LR_TAA_ model described by Shaw et al [29], that uses CSF total tau and Aβ_1−42_ and number APOε4 alleles in a logistic regression model to classify subjects into AD-type CSF and control-like CSF. None of the included subjects was receiving oral, i.v. or i.m. corticosteroids.

### Ethics Statement

Ethics approval was obtained for each institution involved. This study was conducted according to Good Clinical Practice guidelines, the Declaration of Helsinki, US 21CFR Part 50– Protection of Human Subjects, and Part 56– Institutional Review Boards, and pursuant to state and federal HIPAA regulations. Written informed consent for the study was obtained from all subjects and/or authorized representatives and study partners before protocol-specific procedures are carried out. Institutional Review Boards were constituted according to applicable State and Federal requirements for each participating location. The protocols were submitted to appropriate Boards and their written unconditional approval obtained and submitted to Regulatory Affairs at the Alzheimer’s Disease Neuroimaging Initiative Coordinating Center (ADNI-CC) prior to commencement of the study.

### Cognitive Testing

Neuropsychological evaluation and criteria for clinical diagnosis have been described previously [32]. Based on the scores of cognitively normal participants who did not convert to MCI and had a follow-up of at least three years we developed z-scores for processing speed and short term memory as previously described [33].

### Biomarker Collection and Analysis

Details of the CSF collection, processing, storage and measurements as well as the algorithm for classifying subjects as having an AD-like CSF signature were described by Shaw et al [29]. Plasma was prepared from blood samples collected from each study subject, following an overnight fast, at each visit scheduled in the ADNI protocol. At each scheduled visit blood samples were collected, centrifugation, within one hour, placed in dry ice and shipped to the UPenn Biomarker Core laboratory on dry ice. Aliquots (0.5 mL), prepared from plasma samples following thawing at room temperature, were stored in polypropylene aliquot tubes at −80°C until the day of testing. Samples were then interrogated by Rules-Based Medicine, Inc. (RBM, Austin, TX) for levels of 190 analytes using the multiplex Human DiscoveryMAP™ panel and a Luminex 100 platform. Of these, we selected analytes that had less than 10% of the data missing, less than 10% of the data labeled as “LOW” (“LOW” is used by the assay for detected values that are below the least detectable dose) and a coefficient of variation below 20% as outlined in the ADNI statistical analysis plan, which left us with 130 analytes. Assay and procedures are further detailed in the data primer (http://www.adni-info.org/).

Selected analytes had the non-numeric values imputed as follows:

- Values recorded as “LOW” were imputed to least detectable dose/2.
- Values recorded as “>value” were imputed to 2 times the maximum non-missing value for that analyte.
- Missing values were imputed to be the mean of the non-missing values for that analyte.

For each analyte, the distribution of measured values within each diagnostic group was examined. If the distributions were not normal appropriate transformations was applied so the transformed markers approximate normality.

### MRI Processing and Analysis

Acquisition of 1.5-T MRI data at each performance site followed a previously described standardized protocol that was rigorously validated across sites that included a sagittal volumetric 3D MPRAGE with 1.25×1.25 mm in-plane spatial resolution and 1.2-mm thickness for Siemens scanner, 1.09×1.09 mm in-plane spatial resolution and 1.2-mm thickness in General Electric scanner and 1.20×0.94 mm in-plane spatial resolution and 1.2-mm thickness in Philips scanner. Targeted TR and TE values of the ADNI protocol values were TR ∼8.9 ms and ∼3.9 ms, respectively [27].

### Image Analysis

The images were pipeline processed as previously described [34]. The first step was rigid alignment to the ac-pc plane, followed by semi-automated removal of skull and cerebellum tissues. The images were then segmented into four tissue types: grey matter (GM), white matter (WM) sulcal CSF and ventricles (VN). These segmented images were registered to the common brain atlas [35] using high dimensional image warping in order to create RAVENS [34] tissue density maps for GM, WM and VN. The RAVENS maps are the results of elastic registration of original brain regions to the standard template while preserving the original tissue volumes. Therefore, regional volumetric measurements and comparisons are performed via measurements and comparisons of the respective RAVENS maps [36].

### Quantification of Patterns of Atrophy via SPARE-AD

The normalized RAVENS maps were smoothed using 8 mm full-width at half-maximum Gaussian smoothing kernel. With the aim to provide abnormality scores for individual MCI subjects, we utilized a high dimensional pattern classification method [37]. Briefly, this method looks for the combination of brain regions which form a unique pattern that maximally differentiates between two groups. This classifier was trained on the AD and CN subjects in the ADNI [36]; it provides an output that tends to be positive for AD patients and negative for CN subjects.

### MRI Regression Analysis and Group Comparison

For measuring regional correlations between RAVENS and RBM analytes, “beta” maps were created by applying voxel-wise linear regression between baseline RAVENS maps and analytes which were significantly associated with the SPARE-AD score. A voxel-wise t-test was performed to display the different regions on “beta” maps. A threshold corrected for false discovery rate (FDR) was utilized to determine significance of a voxel using AFNI software (http://afni.nimh.nih.gov/afni). Results were tested in a univariate model (analyte concentration and SPARE-AD score) and in multivariate models adjusting for age and clinical diagnosis.

For comparing clinical groups (MCI and AD) adjusting for CSF signature, voxel-based comparisons were done on baseline RAVENS map. Group comparisons involved voxel-by-voxel t-tests applied by the AFNI software. Comparison for multiple corrections utilized the FDR method as implemented in the AFNI software.

### Statistics

SPARE-AD score showed a non-normal distribution; therefore a Yeo-Johnson power transformation was applied [38]. For one-way comparisons of the three clinical groups one way ANOVA test was applied followed by a Tukey HSD for the post-Hoc comparison. For the comparison of categorical variables χ^2^ test was applied. For the comparison of SPARE-AD across clinical and CSF groups a two way ANOVA was applied. Analytes that were significantly associated with SPARE-AD in the model adjusted for age and gender after multiple comparisons were selected for further analyses in two more models [39]. The first model was further adjusted for clinical diagnosis and APOE genotype and the second model included in addition CSF AD-like signature. The errors in the American National Adult Reading test (e-AMNART) [40] were used to assess premorbid performance and used as an estimate of cognitive reserve after a correction using the MMSE score [33], [41]. Normality and homoscedasticity assumptions were tested in all the linear regression models and all of them fulfilled the criteria.

## Results

As expected the CN, MCI and AD groups differed in MMSE and modified ADAS-Cog scores, the percentage of patients with one or more copies of APO ε4 allele and CSF measured analytes. Values are summarized in Table 1.

**Table 1:** Characteristics of subjects included in the study.

	CN (n = 229)	MCI (n = 396)	AD (n = 193)	p-value
Age at exam (years)	75.56	74.42	74.9	0.13
Education (years)	16.03	15.61	14.7	<0.0001
Gender (% male)	52.2%	64.4%	52.8%	0.002
APOE4 (% APOE4 positive)	26.6%	53.3%	65.8%	<0.0001
MMSE	29.11	27.93	23.34	<0.0001
Modified ADAS-Cog	4.00 (1.42)	6.5 (1.52)	8.65 (1.41)	<0.0001
SPARE-AD	−1.40	0.67	1.25	<0.0001
CSF measurements available (%)	50%	50%	53%	
Aβ_42_ (ng/mL)	205.59 (55.09)	162.52 (56.0)	142.98 (40.79)	<0.0001
T-tau (ng/mL)	69.68 (30.37)	101.65 (62.2)	119.15 (59.62)	<0.0001
P-Tau (ng/mL)	24.86 (14.59)	35.64 (18.01)	41.60 (19.79)	<0.0001
Subjects with pathological CSF	30.7%	70.2%	89.2%	<0.0001

### SPARE-AD across Demographic and Clinical Variables, Cognitive Categories and CSF Defined Groups

**Figure 1:**
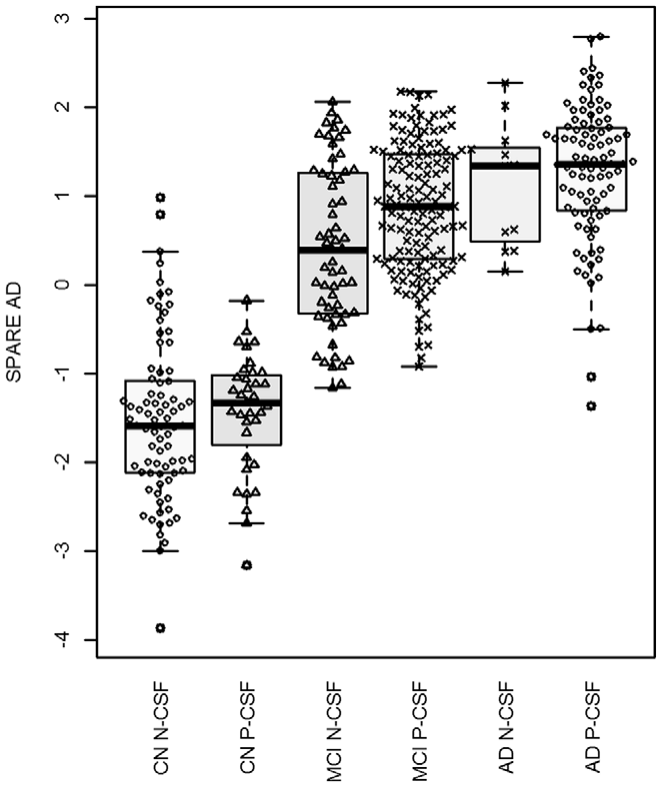
SPARE AD values in the different groups according to clinical diagnosis (CN, MCI, AD) and CSF signature (N-CSF: non AD-like CSF signature; P-CSF: pathological AD CSF signature).

First, we compared the SPARE-AD values across the three clinically defined groups and we found significant differences between these three groups (F (2, 815) = 553.6, p<0.0001). The post-hoc Tukey HSD analysis showed differences between the three groups; CN group showed lower SPARE-AD than MCI (difference (diff) = 1.96, 95% Confidence interval (CI) = 1.79−2.13) and AD groups (diff = 2.65, 95% CI = 2.45−2.85). MCI group showed lower SPARE-AD than the AD group (diff = 0.69, 95% CI = 0.51−0.88). In addition, we added to the model the presence of pathological CSF signature which improved the model (F = 4.48, p = 0.0041). Patients with pathological CSF signature showed lower SPARE AD values (F(1, 408) = 9.17, p = 0.003) (Figure 1).

**Figure 2:**
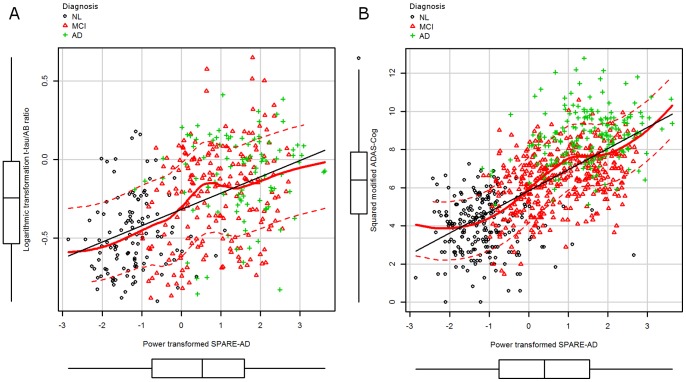
Scatterplot showing log-transformed total-tau/Aβ_42_ ratio and power-transformed SPARE-AD (A) and power transformed SPARE-AD and ADAS-Cog score (B). The marginal box plots on x-axis represent the distribution of the power transformed SPARE-AD and the marginal box-plots on the y-axis represent the t-tau/Aβ_42_ ratio (A) and ADAS-Cog (B) values.

We also found a correlation between the log-scaled t-tau/AB42 ratio and SPARE-AD scores among the whole sample in the analysis adjusted for age, gender and clinical diagnosis (r = 0.66, p<0.0001) (Figure 2A). In a multivariate linear regression model, adjusted for gender, age and re-AMNART, SPARE-AD showed a significant association with the modified ADAS-Cog score (t value (1,799) = 25.1, p<0.0001) (Figure 2B).

We also compared demographic characteristics that have been associated with dementia or changes in MRI measures. Gender showed no association with SPARE-AD (F (1, 815) = 0.57, p = 0.57). Age showed a mild direct correlation with SPARE-AD (r = 0.11, n = 816, p = 0.001). Subjects with one or more APOE4 copies showed lower SPARE-AD values (F (1, 816) = 70.78, p = <0.0001). A small correlation was found between e-AMNART and SPARE-AD (r = 0.14, p<0.001), whereas no correlation was found between SPARE-AD and education (Education: r = −0.035, p = 0.31). We developed a baseline linear regression model to assess the relationship between biomarkers and SPARE-AD. This baseline model, which included clinical diagnosis, CSF signature, APOE, e-AMNART and age, explained 79.4% of the SPARE-AD variability (adjusted-R^2^).

### RBM Plasma Biomarkers and SPARE-AD

After correcting for multiple comparisons, a significant association with SPARE-AD was found for apolipoprotein E (ApoE protein, q<0.0001), brain natriuretic peptide (BNP, q = 0.0007), chromogranin A (CgA, q = 0.033), cortisol (q = 0.033), eotaxin 3(ET-3, q = 0.001), insulin-like growth factor binding protein 2 (IGFBP-2, q = 0.022) and macrophage inflammatory protein1 alpha (MIP-1α, q = 0.031). Adjusted p-values for all the analytes are summarized in Table S1.

We studied these analytes in two more models; in the first one we adjusted for APOE genotype and clinical diagnosis (to avoid findings based on diagnostic group effects) and in the second one we also adjusted for CSF AD-like signature (to avoid findings based on CSF predicted group effects). When we adjusted the previous analytes by APOE genotype the associations between SPARE-AD and CgA, cortisol and IGFBP-2 remained significant (Table 2). In the model adjusted for APOE genotype and CSF AD-like signature only IGFBP-2 and MIP-1α showed an association with SPARE-AD score (Table 3).

**Table 2:** Unstandardized coefficients derived from multiple regression models studying the association between RBM plasma analytes and SPARE AD adjusted for clinical diagnosis, age, and APOE ε4 presence.

	Age	Clinical diagnostic group		APOE	Studied RBM analyte
		MCI	AD		
CgA	0.039 (p<0.001)	1.92 (p<0.001)	2.61 (p<0.001)	0.29 (p = 0.0002)	−0.23 (p = 0.0020)
Cortisol	0.034 (p<0.001)	1.96 (p<0.001)	2.62 (p<0.001)	0.29 (p = 0.0003)	0.81 (p = 0.0048)
IGFBP-2	0.031 (p<0.001)	1.87 (p<0.001)	2.61 (p<0.001)	0.30 (p = 0.0001)	0.59 (p = 0.0016)
MIP1α	0.033 (p<0.001)	1.90 (p<0.001)	2.61 (p<0.001)	0.30 (p = 0.0001)	0.61 (p = 0.13)

**Table 3:** Unstandardized coefficients derived from multiple regression models studying the association between RBM plasma analytes and SPARE AD adjusted for clinical category, APOE ε4 presence, age and CSF signature.

	Age	Clinical diagnostic group		CSF signature	APOE	Studied RBM analyte
		MCI	AD			
CgA	0.030 (p<0.001)	1.84 (p<0.001)	2.35 (p<0.001)	0.32 (p = 0.020)	0.22 (p = 0.052)	−0.14 (p = 0.24)
Cortisol	0.028 (p<0.001)	1.86 (p<0.001)	2.35 (p<0.001)	0.33 (p = 0.017)	0.22 (p = 0.054)	0.32 (p = 0.37)
IGFBP-2	0.023 (p<0.001)	1.78 (p<0.001)	2.35 (p<0.001)	0.29 (p = 0.033)	0.24 (p = 0.032)	0.58 (p = 0.021)
MIP1α	0.024 (p<0.001)	1.75 (p<0.001)	2.30 (p<0.001)	0.36 p = 0.009)	0.22 (p = 0.054)	1.09 (p = 0.028)

### Neuropsychological Measures and Cortisol and MIP-1 α Measurements

We tested the association of CgA, cortisol, IGFBP-2 and MIP-1α with the z-scored cognitive measures (short term memory, delayed memory and processing speed) in a model adjusted for age, clinical diagnostic group and cognitive reserve. Only cortisol showed a significant inverse association with the processing speed (q = 0.045) and a trend with the short term memory domain (q = 0.084). All the other associations were not significant (data not shown).

### Correlation of Plasma Analyte Concentration with MRI Regional Cortical Volumes and CSF Total and p-tau Levels

**Figure 3:**
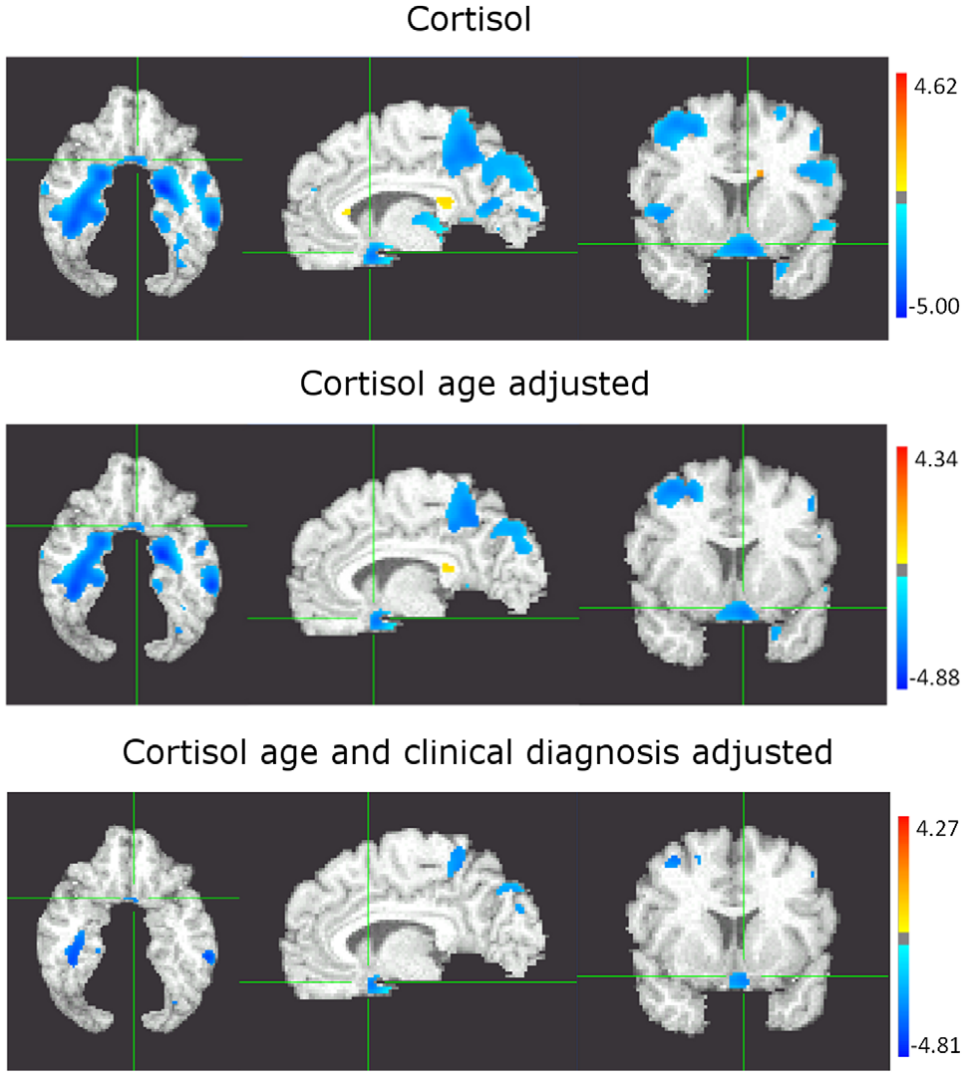
Regression of cortisol without adjustment (A), with adjustment for age (B) and with adjustment for age and clinical diagnosis (C), against regional grey matter (GM) volumes. The color scale represents in blue a decrease in GM volumes associated to a increase levels of the biomarker in plasma. White matter changes (in red) indicate abnormal brain tissue, commonly associated with leukoaraiosis.

In the analysis adjusting for age, subjects with higher cortisol levels showed atrophy in left fusiform gyrus, right dorsolateral prefrontal and ventromedial prefrontal (vmPFC) cortex, biparietal cortex, affecting precuneus and paracentral lobule, and both hipocampi. Changes in the splenium of the corpus callosum associated with white matter changes were also observed. Results were similar although more circumscribed when the analysis was also adjusted for cognitive status (Figure 3).

**Figure 4:**
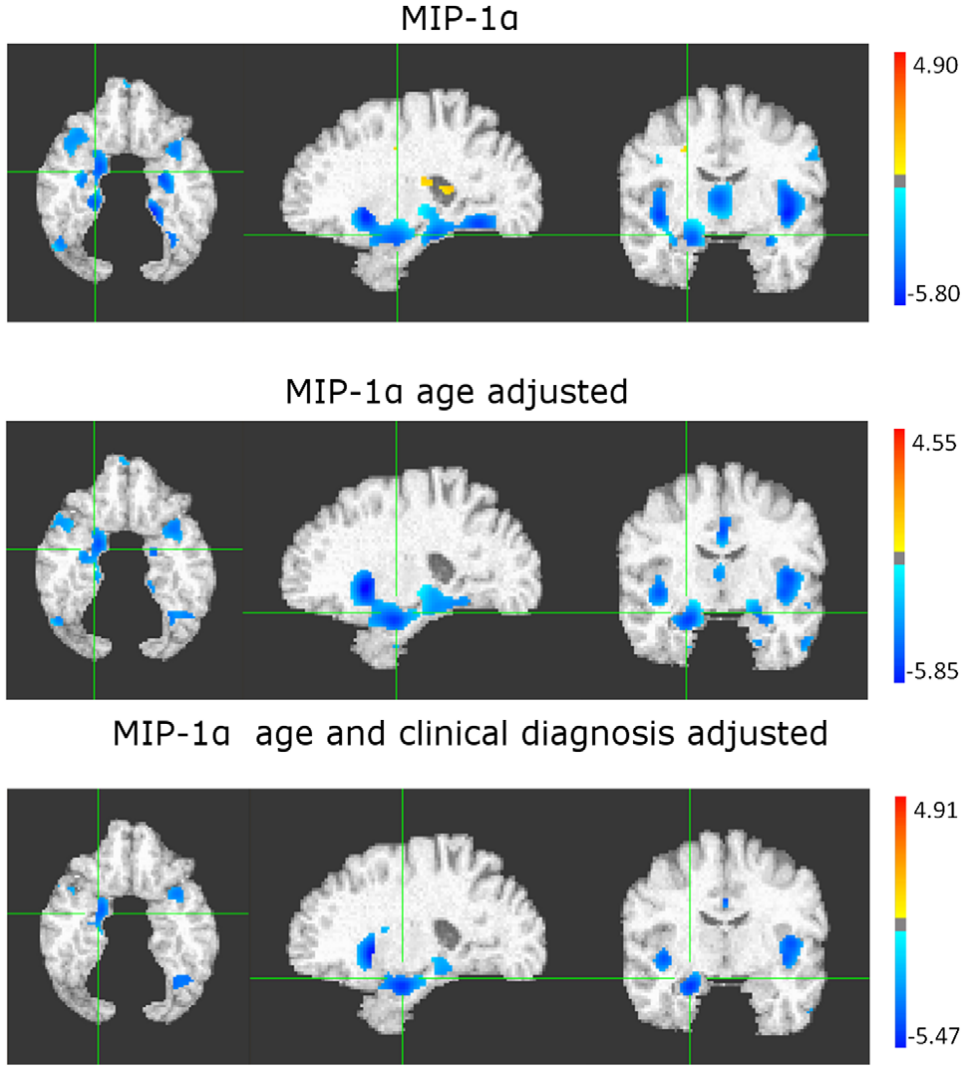
Regression of MIP-1α without (A), with adjustment for age (B) and with adjustment for age and clinical diagnosis (C), against grey matter (GM) volumes. The color scale represents in blue a decrease in GM volumes associated to increased levels of the biomarker in plasma. White matter changes (in red) indicate abnormal periventricular brain tissue, commonly associated with leukoaraiosis.

As shown in Figure 4 subjects with higher MIP-1α after adjustment for age showed bilateral atrophy in the anterior hippocampus, amygdala, insula, as well as in posterior parieto-occipital and occipito-temporal cortex. After adjusting for clinical diagnosis results most of the areas remained significantly associated with MIP-1α (areas not associated with MIP-1α in the last model were the precuneus and occipital cortex).

No association between brain atrophy and the other two plasma biomarkers (CgA and IGFBP-2) were found. However, CgA and IGFBP-2 were associated with CSF total tau levels and CgA was associated in addition with p-tau levels (Table S2).

## Discussion

In this study, we found an association between AD-like patterns of brain atrophy, quantified by the SPARE-AD index, and plasma cortisol, CgA, IGFBP-2 and MIP-1α levels. Increased cortisol levels showed a significant association with worse processing speed and short term memory scores. In addition, we found that SPARE-AD correlates with t-tau/Aβ_42_ ratio and that MCI and AD patients with a negative AD CSF signature have lower (more normal) SPARE-AD values.

We have previously described an association between plasma cortisol levels and PIB PET scores in human subjects [21]. This previous result is in agreement with the results from several animal model studies that have found that chronic stress and chronic increased hypothalamic-pituitary-adrenal axis activity increase Aβ deposition and tau hyperphosphorylation not only in transgenic animal models [42], [43], but also in wild type mice [44] and in-vitro models [45]. Although, some of the studies have described that the effect is only mediated by corticotropin-releasing factor and not by corticosterone [46], [47] other studies have described an effect of glucocorticoids [42], [44], [48], [49]. Some studies indicate that cortisol might independently cause brain atrophy by other pathways that are independent of the amyloid pathway; glucocorticoids have widespread effects on cells through rapid and delayed effects, which are results of no-genomic, indirect genomic and direct genomics effects [50], [51] and increased cortisol levels affect dendritic spine development and reduce neurogenesis [52], [53], [54], [55]. In addition, glucocorticoids and chronic stress modify glutamate transmission in hippocampus and prefrontal cortex [56] and impair long term potentiation [57], [58]. This has been linked to impairment in prefrontal cortex and hippocampus dependent cognitive processes in cognitively normal subjects [59], [60].

One previous study has reported the association between cortisol and MRI changes, describing temporal atrophy with increasing cortisol levels [61]. We found a more widespread pattern of cortisol correlation with brain atrophy that includes dorsolateral prefrontalcortex, vmPFC and insular cortex, as well as cuneus and precuneus in addition to the medial temporal lobe. The dorsolateral prefrontal atrophy and the processing impairment we describe are in line with previous results in animal models that show changes in prefrontal areas and impairments in behavioral tests associated with these areas [53], [62], [63]. Previous studies in aged controls and hypertensive cognitively normal controls had described impairment in prefrontal cortex dependent tasks, but did not include MRI analyses describing the presence of prefrontal cortex atrophy [60], [64]. Lesion in vmPFC impair the retention of extinction learning in animal models [65] and studies in human subjects have also shown an association with fear extinction and vmPFC volume [66]. Chronic exposure to glucocorticoids in animal models leads to an impaired extinction in conditional fear conditioning and has been implicated in altered glutamatergic signaling in vmPFC [67]. We did not have any measures of fear conditioning in our cohort, but our results of atrophy in vmPFC associated to high cortisol levels link cortisol to atrophy in regions related to fear conditioning in human subjects and confirm results described in animal models. As detailed before glucocorticoids regulate dendritic spine development and modulate gene expression resulting in changes that are independent of Aβ deposition and glucocorticoid levels are modulated by chronic stress, therefore part of the associations we described might be related to these factors. Chronic stress and post-traumatic stress disorder studies have described atrophy affecting medial prefrontal cortex, anterior cingulate, right insula and hippocampus [68], [69]. These areas overlap partially with our findings. Therefore, it might be possible that chronic stress has an additional deleterious effect and can be a coincident disease, which is a common finding in aged population of demented subjects [70], [71] or act as an additional risk factor for dementia.

Recent GWAS studies have found susceptibility-linked SNP expressed in immune cells and related to immune response [72], [73], [74], [75], [76] and microglial activation is a common finding in AD neuropathological studies and is involved in the pathological changes [77]. MIP-1α is a ligand for chemokine-CC-motif-receptor 5 (CCR5), a transmembrane-domain, G protein-coupled receptor belonging to the β-chemokine receptor family, which is implicated in the migration of in the migration of monocytes, NK, dendritic and Th1 cells. A previous study has shown that T lymphocytes of AD patients overexpress MIP-1α and the expression of this cytokine is associated with increased transendothelial migration in the endothelial permeability assay and decreased integrity of the human brain microvascular endothelial cell monolayer [78]. The injection of Aβ into ratś hippocampus increased the expression of CCR5 and MIP-1α in endothelial cells and peripheral T cells, respectively [78]. The same researcher described how RAGE was associated to the increased expression of endothelial CCR5, which is mediated by the PI3K and JNK signaling cascade [79]. There are also results that indicate that MIP-1 might be also implicated in the dendritic cell transendothelial migration [80]. Our study further is the first to describe an association with MRI atrophy in human subjects and adds evidence to the involvement of this molecule in Alzheimeŕs disease.

Higher IGFBP-2 levels were associated with increased brain atrophy. This protein has been shown to modulate the actions of IGF-1; high levels result in an inhibition of IGF-1 dependent signaling while low levels increase the signaling [81], [82], [83]. IGF-1signals downstream through insulin receptor substrate 2 (IRS-2) and this pathway is associated with the prenatal and postnatal brain growth [84]. In addition, reduced IRS-2 signaling has been associated with accumulation of phosphorylated tau [85] and non-diabetic AD patients have shown IGF-1 resistance associated with IRS-2 dysfunction [86]. Plasma IGFBP-2 levels correlated with CSF t-tau levels (r = 0.11, p = 0.040) and showed a trend with p-tau levels (r = 0.09, p = 0.075). Therefore, higher IGFBP-2 levels could further impair the already affected IGF-1 signaling in AD cases and results in increased atrophy.

Chromogranins are prohormones, which are the major constituents of secretory large dense-core vesicles. CgA inhibits nicotinic acid transmission and is involved in the regulation of secretory granules. In AD CgA can be found in neuritic plaques and has been implicated in microglial activation [87]. One previous study found low CgA in CSF of patients with early AD [88].

IGFBP-2 and CgA levels were associated with t-tau levels this indicates that their levels might be related to neuronal injury. However, none of these analytes showed a correlation with atrophy in specific brain regions. This might be to the fact that SPARE-AD summarizes the whole pattern of atrophy and increase the power to detect associations due to the avoidance of the multivariate comparisons and adjustments that that are needed for whole brain comparisons.

Only cortisol levels were associated with cognitive measures. There are several factors that could account for the absence of an association between the other plasma biomarkers and the cognitive scores. It is possible the cognitive deficits and the biomarkers take place at different time points, that they show different floor and ceiling effects or that SPARE-AD captures nonlinear relations and measurements from different regions.

Previous studies measuring plasma or serum analytes using similar multi-analyte profiling platforms have been mainly focused on classifying AD patients vs. cognitively normal controls [89], [90], [91], [92], [93]. They have shown a lower [89], [91] or similar classification accuracy [93], [94]. than CSF Aβ_42_, t- and p-tau measurements, indicating that plasma analyte panels might be useful for screening patients and as measure of disease progression [90]. Only one previous study has studied the association between plasma analytes and the classical CSF biomarkers (CSF Aβ_42_, t- and p-tau) finding a different set of analytes [95]. These differences are not unexpected because CSF biomarkers and MRI atrophy behave differently during disease progression [15] and MRI captures changes that might be independent of amyloid and tau deposition as we have proposed.

SPARE-AD also correlated with the CSF T-tau/Aβ_42_ ratio, which discriminates between AD patients and CN individuals [29], distinguishes patients with AD from those with FTLD [96], and predicts future cognitive impairment [97], [98]. While repeated measurements of CSF Aβ as well as PIB PET Aβ plaque burden in AD patients have not been shown to change further with the progression of AD [99], [100], [101], [102], t-tau CSF levels have been reported to increase as AD progresses over time [99], [102] thereby suggesting that the CSF T-tau/Aβ_42_ ratio could increase with the progression of AD and serve as a biomarker of increasing disease severity. The relationship of this ratio with SPARE-AD, which is a measure of brain atrophy that likely reflects the degree of AD neurodegeneration, supports this notion. Furthermore, the moderate correlation with the ADAS-Cog is in agreement with previous findings [26] and consistent with the sensitivity of SPARE-AD to disease progression. The difference in SPARE-AD values in subjects who are positive or negative for the AD CSF signature reflects the ability of the SPARE-AD to identify specific atrophy patterns that are associated with AD pathology. However, the high values in MCI and AD subjects with a negative AD CSF signature indicate that the SPARE-AD may be less specific for other neurodegenerative disorders that cause brain atrophy patterns similar to those in AD. More specific biomarkers can be built by looking specifically at patient populations with different pathologies, as shown before [103], where subtle differences in brain atrophy patterns differentiated between AD and FTD patients.

In summary, our MRI analysis describes for the first time in humans the effect of MIP-1α on cortical areas and the implication in the pathogenesis of AD in agreement with previous results in experimental animals and offers new insight of the association between cortisol and brain atrophy and cognitive changes. The association of brain atrophy with MIP-1α adds further evidence for the importance of the immune response in AD pathogenesis. Last, we also describe an association between our recognition algorithm and CgA and IGFBP-2, which need further validation. These findings shed more light to the association between inflammation markers and AD-like neurodegeneration, a topic of high importance in the aging and AD literatures.

## Supporting Information

Table S1 **Association between plasma analytes and SPARE-AD in the multivariable linear regression analysis adjusted for age and gender.**
(DOCX)

Table S2 **Association between significant plasma analytes and CSF total and p-tau levels in the multivariable linear regression analysis adjusted for age and gender.**
(DOCX)
