# Supplementary material for: Relationship between Plasma Analytes and SPARE-AD Defined Brain Atrophy Patterns in ADNI
Source: PLoS One. 2013 Feb 8;8(2):e55531. doi: 10.1371/journal.pone.0055531 (PMC3568142; doi:10.1371/journal.pone.0055531)
Supplement: Table S1 — Association between plasma analytes and SPARE-AD in the multivariable linear regression analysis adjusted for age and gender. (DOCX) [file pone.0055531.s001.docx]

Supplementary table 1. Association between plasma analytes and SPARE-AD in the multivariable linear regression analysis adjusted for age and gender.

| Analyte | q-value | Analyte | q-value |
| --- | --- | --- | --- |
| Alpha-1-Microglobulin | 0.0878 | Interleukin-13 | 0.7792 |
| Alpha-2-Macroglobulin | 0.2012 | Interleukin-16 | 0.3021 |
| Alpha-1-Antichymotrypsin | 0.2105 | Interleukin-18 | 0.5047 |
| Alpha- 1 Antitrypsin | 0.1682 | Interleukin-3 | 0.7894 |
| Angiotensin-Converting Enzyme | 0.3021 | Interleukin-6 receptor | 0.2105 |
| Alpha-Fetoprotein | 0.7715 | Interleukin-8 | 0.7715 |
| Agouti-Related Protein | 0.3796 | Insulin | 0.0878 |
| Angiopoietin-2 | 0.3021 | Interferon gamma Induced Protein 10 | 0.6764 |
| Angiotensinogen | 0.244 | Kidney Injury Molecule-1 | 0.7894 |
| Apolipoprotein A-I | 0.196 | Leptin | 0.1645 |
| Apolipoprotein A-II | 0.1093 | Luteinizing Hormone | 0.7894 |
| Apolipoprotein A-IV | 0.4074 | Apolipoprotein(a) | 0.7894 |
| Apolipoprotein B | 0.4103 | Monocyte Chemotactic Protein 1 | 0.655 |
| Apolipoprotein C-I | 0.394 | Monocyte Chemotactic Protein 2 | 0.733 |
| Apolipoprotein C-III | 0.3568 | Monocyte Chemotactic Protein 3 | 0.6989 |
| Apolipoprotein D | 0.7894 | Monocyte Chemotactic Protein 4 | 0.7894 |
| Apolipoprotein E | <0.0001 | Macrophage Colony-Stimulating Factor 1 | 0.7715 |
| Apolipoprotein H | 0.6926 | Macrophage Derived Chemokine | 0.7894 |
| AXL Receptor Tyrosine Kinase | 0.7894 | Macrophage Migration Inhibitory Factor | 0.7995 |
| Beta-2 Microglobulin | 0.2858 | Monokine Induced by Gamma Interferon | 0.733 |
| Brain-Derived Neurotrophic Factor | 0.7894 | Macrophage Inflammatory Protein-1 alpha | 0.0309 |
| B Lymphocyte Chemoattractant | 0.3021 | Macrophage Inflammatory Protein-1 beta | 0.7894 |
| Bone Morphogenetic Protein 6 | 0.1651 | Macrophage Inflammatory Protein-3 alpha | 0.4074 |
| Brain Natriuretic Peptide | 0.0007 | Matrix Metalloproteinase-1 | 0.2065 |
| Betacellulin | 0.1651 | Matrix Metalloproteinase-10 | 0.7894 |
| Comement C3 | 0.7715 | Matrix Metalloproteinase-2 | 0.4074 |
| Cancer Antigen 19-9 | 0.1093 | Matrix Metalloproteinase-7 | 0.7762 |
| Calcitonin | 0.7894 | Myeloid Progenitor Inhibitory Factor 1 | 0.7995 |
| CD40 antigen | 0.4798 | Myeloperoxidase | 0.4074 |
| CD40 Ligand | 0.7894 | Myoglobin | 0.5352 |
| CD5 | 0.3211 | Neutrophil Gelatinase-Associated Lipocalin | 0.2684 |
| Carcinoembryonic Antigen | 0.3021 | Neuronal Cell Adhesion Molecule | 0.344 |
| Chromogranin-A | 0.0332 | Osteopontin | 0.7894 |
| Creatine Kinase-MB | 0.1287 | Plasminogen Activator Inhibitor 1 | 0.7894 |
| Clusterin | 0.6547 | Prostatic Acid Phosphatase | 0.4103 |
| Ciliary Neurotrophic Factor | 0.733 | Pulmonary and Activation-  Regulated Chemokine | 0.733 |
| Comement Factor H | 0.6645 | Platelet-Derived Growth Factor BB | 0.7894 |
| Cortisol | 0.0332 | Placenta Growth Factor | 0.1682 |
| C-peptide | 0.244 | Pancreatic Polypeptide | 0.0646 |
| C-Reactive Protein | 0.0878 | Prolactin | 0.7993 |
| Cystatin-C | 0.6547 | Proinsulin, Intact | 0.4074 |
| Epidermal Growth Factor | 0.6764 | Proinsulin, Total | 0.733 |
| Epidermal Growth Factor Receptor | 0.7894 | Peptide YY | 0.0878 |
| Epithelial-Derived Neutrophil-  Activating Protein 78 | 0.6989 | Receptor for advanced glycosylation  end products | 0.7935 |
| Eotaxin-1 | 0.6764 | T-Cell Specific Protein RANTES | 0.7715 |
| Eotaxin-3 | 0.001 | Resistin | 0.5726 |
| E-Selectin | 0.4788 | Serum Amyloid P-Component | 0.7894 |
| Fatty Acid Binding Protein, heart | 0.7894 | Stem Cell Factor | 0.7715 |
| Factor VII | 0.389 | Serum Glutamic Oxaloacetic Transaminase | 0.244 |
| FASLG Receptor | 0.7709 | Sex Hormone-Binding Globulin | 0.0878 |
| Fas Ligand | 0.1666 | Sortilin | 0.7894 |
| Fetuin-A | 0.7935 | Thyroxine-Binding Globulin | 0.1093 |
| Fibroblast Growth Factor 4 | 0.7894 | Trefoil Factor 3 | 0.7894 |
| Fibrinogen | 0.4074 | Thrombospondin-1 | 0.6989 |
| Ferritin | 0.4074 | Tissue Inhibitor of Metalloproteinases 1 | 0.0878 |
| Follicle Stimulating Hormone | 0.1279 | Tenascin-C | 0.0999 |
| Growth Hormone | 0.2105 | Tumor Necrosis Factor Receptor-Like 2 | 0.1666 |
| Growth-Regulated alpha protein | 0.7894 | Thrombopoietin | 0.733 |
| Glutathione S-Transferase alpha | 0.1651 | TNF-Related Apoptosis Inducing  Ligand Receptor 3 | 0.7762 |
| Haptoglobin | 0.7491 | Serotransferrin | 0.2266 |
| Chemokine CC-4 | 0.2684 | Thyroid Stimulating Hormone | 0.7894 |
| Hepatocyte Growth Factor | 0.3559 | Transthyretin | 0.0512 |
| T Lymphocyte-Secreted Protein I-309 | 0.7894 | Vascular Cell Adhesion Molecule-1 | 0.0878 |
| Immunoglobulin A | 0.4458 | Vascular Endothelial Growth Factor | 0.2012 |
| Insulin-like Growth Factor-Binding Protein 2 | 0.0217 | Vitronectin | 0.1093 |
