# Supplementary material for: Relationship between Plasma Analytes and SPARE-AD Defined Brain Atrophy Patterns in ADNI
Source: PLoS One. 2013 Feb 8;8(2):e55531. doi: 10.1371/journal.pone.0055531 (PMC3568142; doi:10.1371/journal.pone.0055531)
Supplement: Table S2 — Association between significant plasma analytes and CSF total and p-tau levels in the multivariable linear regression analysis adjusted for age and gender. (DOCX) [file pone.0055531.s002.docx]

Supplementary table 2. Association between significant plasma analytes and CSF total and p-tau levels in the multivariable linear regression analysis adjusted for age and gender.

| Plasma analyte | Association with CSF total tau | Association with CSF p-tau |
| --- | --- | --- |
| CgA | 0.002 | 0.012 |
| Cortisol | 0.062 | 0.11 |
| IGFBP-2 | 0.018 | 0.17 |
| MIP1α | 0.41 | 0.62 |

p-values
